# Modeling and Control of Adjustable Articulated Parallel Compliant Actuation Arrangements in Articulated Robots

**DOI:** 10.3389/frobt.2018.00004

**Published:** 2018-02-12

**Authors:** Wesley Roozing

**Affiliations:** ^1^Department of Advanced Robotics, (Fondazione) Istituto Italiano di Tecnologia, Genova, Italy

**Keywords:** compliant joints, force/torque control, series-parallel elastic actuation, energy efficient actuation, articulated robots

## Abstract

Considerable advances in robotic actuation technology have been made in recent years. Particularly the use of compliance has increased, both as series elastic elements as well as in parallel to the main actuation drives. This work focuses on the model formulation and control of compliant actuation structures including multiple branches and multiarticulation, and significantly contributes by proposing an elegant modular formulation that describes the energy exchange between the compliant elements and articulated multibody robot dynamics using the concept of power flows, and a single matrix that describes the entire actuation topology. Using this formulation, a novel gradient descent based control law is derived for torque control of compliant actuation structures with adjustable pretension, with proven convexity for arbitrary actuation topologies. Extensions toward handling unidirectionality of elastic elements and joint motion compensation are also presented. A simulation study is performed on a 3-DoF leg model, where series-elastic main drives are augmented by parallel elastic tendons with adjustable pretension. Two actuation topologies are considered, one of which includes a biarticulated tendon. The data demonstrate the effectiveness of the proposed modeling and control methods. Furthermore, it is shown the biarticulated topology provides significant benefits over the monoarticulated arrangement.

## 1 Introduction

Recent years have seen a paradigm shift in the field of robotic actuation from stiff, mainly position controlled concepts to compliant actuators in force control. This increased focus on use of compliance has taken place by addition of elastic elements both in series with servo drives, and in parallel to the main actuation of robotic systems. Many of the proposed concepts take inspiration from biological systems, in both their topology as well as the capacity for energy storage and release during motion. In robotic systems, they provide significant further benefits such as improved force control performance and physical robustness against impacts.

Out of the concepts proposed in literature, compliance in series with the actuation drive, known as series elastic actuation (SEA) and pioneered by Pratt in the 1990s (Pratt and Williamson, 1995), has been the most widely adopted. SEAs have evolved to become the core component of nearly all articulated robots. Addition of compliant elements in parallel to the main actuation drives, known as parallel elastic actuation (PEA), has seen less adoption than SEA. However, their benefits have been repeatedly demonstrated, particularly in terms of energy efficiency: in actuator test bench setups (Mettin et al., 2010; Haeufle et al., 2012; Mathijssen et al., 2015, 2016; Plooij et al., 2016), hopping robots (Liu et al., 2015), bipedal walkers (Yang et al., 2008; Mazumdar et al., 2016), and humanoids (Shirata et al., 2007). Another field of application is that of prostheses, where parallel compliance has been utilized in prosthetic ankles (Au et al., 2009; Realmuto et al., 2015; Jimenez-Fabian et al., 2017) and knees (Rouse et al., 2013; Pfeifer et al., 2015), to reduce the motor torque required to produce the desired deflection-torque profiles.

A common challenge with parallel compliance is that during some stages of the motion the torque generated by the parallel element does not correspond well to the desired torque on the joint. The result of this is that the main actuation drive has to work against the parallel compliance in order to obtain the desired joint torque or motion. To address this, many works employ unidirectional elements (Au et al., 2009; Mettin et al., 2010; Realmuto et al., 2015; Mazumdar et al., 2016; Jimenez-Fabian et al., 2017), clutches/switches (Haeufle et al., 2012; Rouse et al., 2013; Liu et al., 2015; Plooij et al., 2016), secondary motors to change the pretension (Mathijssen et al., 2015, 2016; Roozing et al., 2015, 2016), or a combination of these concepts to engage and disengage the parallel elements at desired moments.

Many biological systems have been found to contain biarticulated muscle structures, where a single muscle spans multiple joints. The human body incorporates many biarticular muscles; for example, the rectus femoris and hamstrings, which span the hip and knee joints as an antagonistic pair, the biceps that spans the shoulder and elbow, and the gastrocnemius muscle, which spans the knee and ankle joints. In the field of biomechanics, biarticulated muscles have been identified to transfer mechanical power between joints (Schenau, 1989; van Soest et al., 1993; Prilutsky and Zatsiorsky, 1994), used, for example, to greatly increase jumping height.

Considering the benefits demonstrated in biological systems, several authors have sought to employ multiarticulated actuation in articulated robots. In such contexts, motor drives and elastic elements that drive the joints of a robotic system are sometimes referred to as (actuation) branches. In Klein and Lewis (2009), the transfer of mechanical power between joints was experimentally demonstrated in a leg that models all nine major muscle groups in the human lower limb in the saggital plane. In Iida et al. (2008) and Niiyama et al. (2007), biarticulation was used in walking and jumping, respectively. Salvucci et al. (2014) showed how biarticulation can improve the end-effector force ellipsoid. The recently introduced compliant bipedal walker (Loeffl et al., 2016) also included a biarticulated tendon spanning ankle and knee. Babič et al. (2009) showed the benefits of a biarticulated compliant tendon spanning the ankle and knee joints in terms of jumping height through optimized motions of—and experiments with—a jumping robot.

In Tsagarakis et al. (2014) and Roozing et al. (2015, 2016), a 1-DoF leg prototype was designed that combines a high power SEA main drive with a parallel compliant high efficiency energy storage branch with adjustable pretension using a secondary motor. Using a novel distributed controller that actively utilizes both branches, the authors experimentally verified the potential of both mechanism and controller, demonstrating a 65% reduction in electrical power consumption when compared to conventional SEA, while performing cyclic squatting motions. The concept, its design optimization and control methods were generalized to multi-DoF systems and biarticulated actuation configurations in Roozing et al. (2016). Simulation studies performed on a 2-DoF leg demonstrated significant improvements in electrical energy efficiency and reduction in peak torque and electrical power requirements, compared to SEA only, while performing elliptical trajectories with the hip in a squatting motion. A biarticulated actuation arrangement was shown to further improve energy efficiency, compared to an arrangement utilizing solely monoarticulated parallel compliance.

This article builds upon these existing concepts and focuses on the model formulation and control of compliant actuation arrangements including multiple branches and multiarticulation, and contributes by:

- Proposing a modular formulation that describes the energy exchange between the compliant elements and articulated multibody robot dynamics using power flows and a single matrix that describes the entire actuation topology.
- Using this formulation to derive a novel gradient descent based control law for compliant actuation structures with adjustable pretension, with proven convexity for arbitrary actuation topologies.

This article is structured as follows. Section 2 builds up the proposed model formulation, starting at single-joint, single-branch systems and expanding into multi-DoF, multiactuator systems with multiarticulation. Section 3 briefly discusses the design optimization method originally presented in Roozing et al. (2016), followed by the proposed control strategies and an illustrative example in Section 4. A simulation study to validate the proposed methods is presented in Section 5, followed by concluding remarks and suggestions for future work in Section 6.

## 2 Compliant Actuation

In general, the torque *τ* generated on a single joint with configuration *q* by a single compliant tendon can be written as

(1) τ(q)=−k n(p+n q),

where *k* denotes the linear tendon stiffness, *n* denotes the transmission ratio, and *p* denotes the pretension position, or the position where element is at rest length. The sign of *n* indicates the direction of *q* that increases the elongation of the tendon. The elongation Δ of the element is thus given by

(2) Δ=p+n q.

In implementations of elastic elements with high energy storage, unidirectional elements are often used, such as those constructed of natural rubber, usable in elongation and not in compression. For those, the torque is thus dependent on the sign of Δ:

(3) $$\tau \left(q\right)=\left\{\begin{array}{cc} -k\;n\Delta & \Delta >0\\ 0 & \text{Otherwise}. \end{array}\right.$$

We will explicitly take this property into account in the synthesis of our control strategies in Section 4.

### 2.1 Adjustable Parallel Compliance

**Figure 1:**
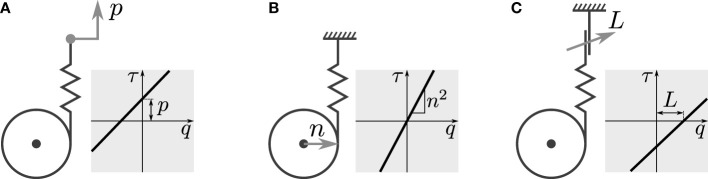
Types of adjustability: **(A)** pretension, **(B)** transmission ratio, and **(C)** engagement mechanism.

While parallel compliance can provide many benefits, the parallel branches may not be continuously required, nor may their static properties be suitable for every task or configuration required of the robot. In these cases, adjustability is a desirable property of the parallel branches, that may be exploited to further increase the effectiveness of the system. In general, for the compliant arrangements considered here, three parameters may be considered for adjustment: pretension, transmission ratio(s), and engageability. Generally, stiffness of mechanical elastic elements cannot be adjusted directly; instead, adjustable transmission ratio is commonly utilized. Figure 1 gives a graphical overview of the three types of adjustability. Adjustable pretension can in some sense be considered series-elastic actuation; however, in contexts where the stiffness value is relatively small and the compliance augments some main drive, this is commonly referred to as parallel compliance with adjustable pretension.

Each method has its respective benefits and drawbacks:

- Pretension and transmission ratio can be continuously adjusted, which is beneficial for many control strategies.
- Adjustment of the transmission ratio allows to completely disengage the branch, assuming the ratio can reach zero. However, as this method changes slope and not offset, it cannot provide nonzero torques at the joint configuration corresponding to the elastic element’s equilibrium position. Furthermore, mechanical implementation of variable transmission ratio is often cumbersome.
- Clutch mechanisms are simple to realize, however, their disengagement can be problematic due to release of stored energy, when one side contains an elastic element under tension (as in this case).

Due to the binary nature of clutch mechanisms, we shall focus on the other two, namely adjustable pretension and adjustable transmission ratio. We consider the impact of these methods of adjusting compliance properties on generated torque, by returning to (1). For adjustable pretension, we take the derivative w.r.t. *p*:

(4) $$\frac{\delta \tau }{\delta p}=-k\;n,$$

which does not depend on *p*, showing the adjustment is linear, and is independent of *q*, i.e., changing *p* results in a constant offset of *τ*. For adjustable transmission ratio, we take the derivative w.r.t. the transmission ratio *n*:

(5) $$\frac{\delta \tau }{\delta n}=-k\;p-2\;k\;n\;q,$$

which is a function of *n*, hence the adjustment is not linear. It can be observed the first term results in a change in offset of *τ* for *p* ≠ 0, and the second term shows that the change of slope of *τ* (*q*) scales with 2 *n*. As noted before, for *n* = 0 → *τ* = 0, allowing to effectively disengage the compliant element.

### 2.2 Multiarticulation

**Figure 2:**
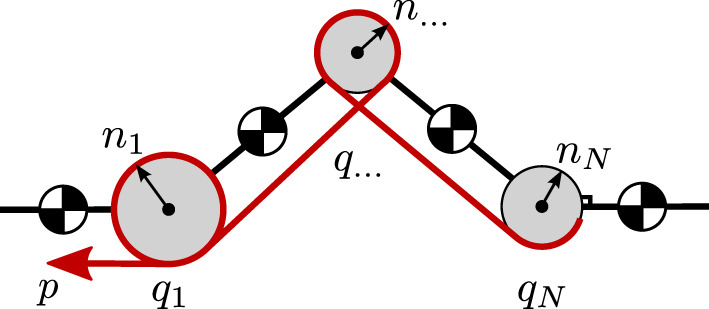
Multiarticulation of a single tendon for an *N*-joint kinematic chain. The elastic tendon with pretension position *p* and stiffness *k* is shown in red.

In this section, we formulate multiarticulated compliant branches, that span any number of joints. Assuming an articulated robot with *N* joints, and a configuration vector given by **q** = [*q*_1_, *q*_2_, … , *q_N_*]*^T^* ∈ Q where the joint space $Q\subset R^{N}$, the deflection Δ∈R of a single multiarticulated branch is given by

(6) $$\Delta =p+n_{1}\;q_{1}+n_{2}\;q_{2}+\cdots +n_{N}\;q_{N},$$

where $n_{1}\ldots n_{N}\in R$ denote the transmission ratios for each of the *N* joints, shown also in Figure 2. Again, the sign of each *n_i_* indicates the direction of the corresponding joint *q_i_* that increases the elongation of the tendon.

The torque $\tau_{i}\in R$ applied to the *i*th joint can then be written as

(7) $$\tau_{i}=-k\;n_{i}\left(p+n_{1}\;q_{1}+n_{2}\;q_{2}+\cdots +n_{N}\;q_{N}\right),\;i=1\ldots N,$$

where *k* denotes the stiffness of the branch. Contracting the transmission ratios into vector form, we can write the torque $\tau \in R^{N}$ applied to all *N* joints as

(8) $$\tau =-{\text{t}}^{T}k\left(p+\text{t q}\right)\in R^{N},$$

where the row vector $\text{t}=\left[n_{1},n_{2},\ldots ,n_{N}\right]\in R^{N}$ both maps the joint configurations to elastic element elongation, and maps the produced linear tendon force back to joint torques. The deflection is written using **t** as Δ = *p* + **t q**, and the linear tendon force f∈R is equal to *f* = *k* (*p* + **t q**).

In terms of adjustability of multiarticulated configurations, adjusting *p* affects the torque on all joints linearly:

(9) $$\nabla_{p}\tau =-{\text{t}}^{T}k\in R^{N},$$

whereas adjusting the transmission ratios **t** affects joints nonlinearly and is also dependent on **q**:

(10) $$\nabla_{\text{t}}\tau =-k{\left(\text{q t}\right)}^{T}-k\;I\left(p+\text{t q}\right)\in R^{N\times N},$$

where *I* denotes the N × N identity matrix. It can be observed the first term arises from the change in elongation of the element due to the changed transmission ratio, and the second diagonal term arises from the change in conversion ratio from linear tendon force to torque on the joints.

### 2.3 Multiple Branches

In this section, we expand the previous section to a unified formulation for multiple, possibly multiarticulated branches. Supposing we have *M* parallel elastic branches, we gather all their respective **t** vectors in an *actuation topology* matrix $T\in R^{M\times N}$, that fully describes the actuation topology:

(11) $$T=\left[\begin{array}{c} {\text{t}}_{1}\\ \ldots \\ {\text{t}}_{M} \end{array}\right],$$

which gives rise to the vector of deflections: $\Delta =\text{p}+T\text{ q}\in R^{M}$, and correspondingly the total torque $\tau \in R^{N}$ on the robot exerted by the branches:

(12) $$\tau =-\;T^{T}\;K\left(\text{p}+T\text{ q}\right),$$

where $K\in R^{M\times M}$ is the diagonal matrix of stiffness values. Note that throughout this article superscript [⋅]*^T^* denotes transpose, whereas *T* denotes the matrix. The vector of linear tendon forces $\text{f}\in R^{M}$ follows as **f** = *K* (**p** + *T* **q**). Similar to the single branch case, adjusting the pretensions **p** for *M* actuators yields:

(13) ∇pτ=−TT K∈RN×M.

For adjustable transmission ratios, calculating $\nabla_{T}\;\tau$ yields a 3D tensor, of which the components for the *m*th actuator are given by

(14) $$\nabla_{{\text{t}}_{m}}\;\tau =-k_{m}\;{\left(\text{q}{\text{ t}}_{m}\right)}^{T}-k_{m}\;I\left(p_{m}+{\text{t}}_{m}\text{ q}\right)\in R^{N\times M}.$$

Considering each **t***_m_* is of dimension *N*, this means that up to *N M* variables are involved. Of course, usually *T* can be considered quite sparse since all tendons are not driving all joints.

In both cases, the gradient with respect to the joint configurations is:

(15) $$\nabla_{\text{q}}\;\tau =-T^{T}K\;T\in R^{N\times N}.$$

Stopping for a moment to consider the different dynamics of adjustable pretension and adjustable transmission of multiarticulated compliance, we find the latter arguably provides more freedom in shaping the provided torque than the former, due to changing the slope and the larger number of degrees of freedom (in multiarticulation). As aforementioned, this also adds the potential benefit of disengaging elements entirely from desired joints. However, significant drawbacks exist due to the nonlinear behavior on a potentially much larger configuration space, combined with increased complexity in realizing such structures. Therefore, at this point, we choose to focus on adjustable pretension in our modeling and control formulation.

We now proceed with a modular model formulation using energy exchange through the concept of power ports. Taking the time derivative of the deflections **Δ**, we find the rate of change of the deflection of the elastic elements is given by

(16) $$\dot{\Delta }=\dot{\text{p}}+T\;\dot{\text{q}}.$$

Given that the power flow into an elastic element is given by the force multiplied by rate of displacement (i.e., $P=f\;\dot{\Delta }$), we find from port-Hamiltonian theory that $\left(\text{f},\dot{\Delta }\right)\in R^{N}$ and $\left(\tau ,\dot{\text{q}}\right)\in R^{N}$ describe an *N*-dimensional power port that exchanges energy between the rigid body robot and compliant actuation branches driving it. This power flow is the sum of each of the power flows in/out of the individual elastic elements; indeed, power may flow between the elastic elements as well.

**Figure 3:**
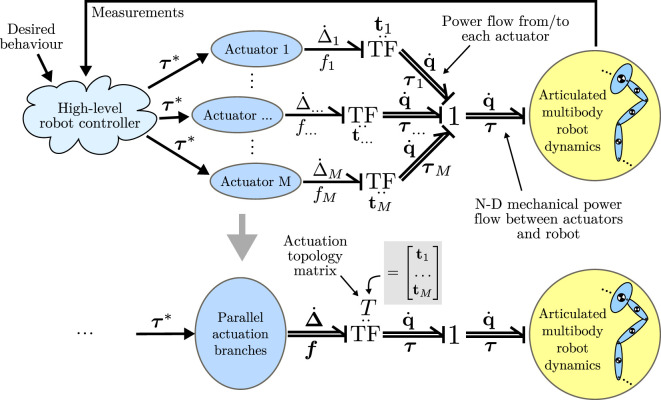
Model formulation using *N*-dimensional power ports, shown in Bond graph notation. The bottom diagram shows how the actuation topology matrix *T* describes the power flow between actuators and robot.

This concept is depicted graphically in Figure 3, using Bond graph notation. The first diagram shows the notation using **t** vectors, and the bottom diagram shows how the *T* matrix completely describes the power flow between actuators and robot. This formulation has several advantages for rapidly evaluating different actuation topologies; by simply modifying *T* the transmission ratios and actuation configuration of tendons can be quickly modified. It also enables modularity of the modeling and simulation procedures by separating actuator dynamics from the articulated multibody dynamics of the robot.

## 3 Optimization of Design Parameters

In this section, we briefly discuss the optimization of design parameters presented in Roozing et al. (2016). Based on gravitational load and inertial properties, the compliance design parameters can be chosen to achieve desired compensation torque over the joint workspace, resulting in higher energy efficiency and reduction of peak torque/power requirements on the main joint actuators. The optimization procedure considers the transmission ratios contained in *T*, elastic element stiffness values contained in *K*, and pretension positions in **p** as optimization variables. We first define the error vector **e***_d_* for a leg configuration **q** as

(17) $${\text{e}}_{d}\left(\text{q},\varphi \right)=\tau \left(\text{q},\varphi \right)+\zeta \left(\text{q}\right)\in R^{N},$$

where ***τ***(**q**, ***φ***) denotes the net tendon torques (12) acting on the leg DoFs, and ***φ*** contains the considered design parameters *T*, *K* and **p**. The function ζ(q) denotes the vector function of desired torques; here, we consider gravitational joint torque compensation, ***ζ***(**q**) = **g**(**q**). For highly dynamic systems for which the desired dynamic behavior is known at design time, ***ζ*** can be chosen to include inertial, Coriolis, and damping components for efficient execution of those motions. This is done by designing the dynamic behavior and then obtaining the required actuation torques during each phase of the task through inverse dynamics, thus obtaining ***ζ*** (**q**). It is also possible to optimize for multiple tasks simultaneously by obtaining ***ζ*** (**q**) as a weighted linear combination of multiple tasks. Here, however, we consider the desired motions not to be known at design time.

The total error $E_{d}\left(\varphi \right)\in R$ is defined as the integrated *l*^2^-norm over a subset of the workspace:

(18) $$E_{d}\left(\varphi \right)=\int_{\text{q}\in Q_{d}}∥{\text{e}}_{d}\left(\text{q},\varphi \right){∥}_{2}\;d\text{q},$$

where $Q_{d}\subset Q$ is a subset of the joint workspace on which to optimize and depends on the specific robot. For robots for which the desired trajectories are known *a priori*, $Q_{d}$ can be set to this trajectory in joint space. Using the *l*^2^-norm approximates minimizing the electrical power consumption directly, as the electrical power of a BLDC motor can be approximated (neglecting back-EMF and electrical dynamics) by its squared torque. An optimal solution ***φ**_opt_* minimizes *E_d_*:

(19) $$\begin{array}{l} \varphi_{\mathrm{opt}}=\underset{\varphi }{\text{min}}\;E_{d}\left(\varphi \right)\\ \;\text{s}.\text{t}.\underline{\varphi }\leq \varphi \leq \bar{\varphi }, \end{array}$$

where $\underline{\varphi }$ and $\bar{\varphi }$ denote the lower and upper bounds of ***φ***, respectively. Note that this design optimization procedure includes **p** into the optimization as a parameter. As such, it attempts to optimize the design such that it provides the desired torques over the joint space as accurately as possible without pretension adjustment. The control strategies presented in the next section exploit the fact that pretension is adjustable, which can be used to further increase efficiency of such systems.

## 4 Control Strategies

Various control strategies can be employed to effectively utilize adjustability of (parallel) compliance. In Roozing et al. (2015) and Roozing et al. (2016), inversion of the peractuator pretension–torque relations was utilized to obtain the pretension position references that lead to the desired torques. To handle coupling resulting from multiarticulation, the equations were solved in a cascaded manner. However, this method generalizes poorly for arbitrarily complex structures and requires a degree of designer intuition.

In the following sections, we propose two alternative methods to solve the torque control problem through adjustable pretension, employing the multi-DoF, multiactuator formulation of Section 2.3. The first relies on the (pseudo)inverse of the topology matrix *T*, which is a generalization of the previous method. We show this method suffers from limitations in certain situations, with regards to coupling and unidirectionality of elastic elements. The second relies on gradient descent, which allows to simultaneously take coupling and unidirectionality of the elastic elements as well as achievable pretension adjustment speeds into account.

### 4.1 (Pseudo)inverse

Returning to the multi-DoF, multiactuator torque equation (12), we observe that it can be solved for **p**:

(20) $${\text{p}}^{*}=-{\left(T^{T}K\right)}^{-1}\tau^{*}-T\text{ q},$$

where τ* denotes the desired torque, and p* denotes the resulting desired pretension positions, respectively. If *T* is not full rank, the pseudoinverse may be used in (20). This method is suitable for position controlled pretension as in Roozing et al. (2016), and is a multi-DoF generalization of the method presented in that work. However, using the (pseudo)inverse, it is not possible to take unidirectionality of elastic elements into account. Suitable preprocessing of the desired torque vector can resolve this issue in certain cases, however, this is not a general solution, hence this method is feasible only if the resulting **Δ** ≥ 0 or if bidirectional elastic elements are used.

### 4.2 Gradient Descent

To obtain a gradient descent based torque control law, we start with the torque control error **e**, defined as $\text{e}=\tau^{*}-\tau \in R^{N}$. Taking the gradients with respect to **p** and **q**, we obtain the rate of change of **e** as

(21) $$\dot{\text{e}}=\left(\nabla_{\text{p}}\text{e}\right)\dot{\text{p}}+\left(\nabla_{\text{q}}\text{e}\right)\dot{\text{q}},$$

where for now we have assumed the use of bidirectional elastic elements, or equivalently, **Δ** ≥ **0**, i.e., no branches are in slack. Section 4.2.1 introduces an extension for when this assumption does not hold. Since we can assume that the desired joint torques do not depend on **p**, we have $\nabla_{\text{p}}\;\tau^{*}=\text{0}$, and the above equation can be rewritten using the definition of **e** as

(22) $$\dot{\text{e}}=-\left(\nabla_{\text{p}}\;\tau \right)\dot{\text{p}}+\left(\nabla_{\text{q}}\;\tau^{*}-\nabla_{\text{q}}\;\tau \right)\dot{\text{q}},$$

where $\nabla_{q}\;\tau^{*}$ depends on high-level controller and robot dynamics, and from Section 2.3 we recall:

(23) $$\begin{array}{l} \nabla_{\text{p}}\;\tau =-T^{T}K,\\ \nabla_{\text{q}}\;\tau =-T^{T}K\;T. \end{array}$$

At this point, we introduce the squared *l*_2_-norm of **e** as our error measure. Using the results above, the chain rule, and $\nabla ∥\text{e}{∥}_{2}^{2}=2\text{ e}$, we compute the gradient with respect to **p**:

(24) $$\begin{array}{lll} \nabla_{\text{p}}{∥\text{e}∥}_{2}^{2} & = & \left(\nabla_{\text{p}}\text{e}\right)\;\nabla {∥\text{e}∥}_{2}^{2}\\ & = & 2{\left(T^{T}K\right)}^{T}\left(\tau^{*}-\tau \right). \end{array}$$

Setting rate of change of **p** as $\dot{\text{p}}=-\gamma_{e}\;\nabla_{\text{p}}{∥\text{e}∥}_{2}^{2}$, where 0 < *γ_e_* ≤ 1 is a suitable scaling constant, ensures asymptotic convergence of **e** given $\dot{\text{q}}=\text{0}$; Section 4.2.2 discusses the extension to $\dot{\text{q}}\neq \text{0}$. Furthermore, note that (24) does not depend on $\nabla_{\text{q}}\;\tau^{*}$, i.e., the controller is independent of the specific robot dynamics or its high-level controllers.

By taking the second-order gradient of the squared *l*_2_-norm of **e**, we show that it is globally convex, and thus **e** converges to the global minimum:

(25) $$\begin{array}{lll} \nabla_{\text{p}}^{2}{∥\text{e}∥}_{2}^{2} & = & \nabla_{\text{p}}\left[2{\left(T^{T}K\right)}^{T}\left(\tau^{*}-\tau \right)\right]\\ & = & 2{\left(T^{T}K\right)}^{T}\left(T^{T}K\right), \end{array}$$

which is positive definite as the quadratic form is always positive definite. This proves global asymptotic convergence of the error.

#### 4.2.1 Constraint

The previous section assumed that either bidirectional elastic elements were used, or equivalently, unidirectional elements for which the elongation **Δ** ≥ **0**. This section adds a dynamic potential function of which we take the gradient, so that the control algorithm will never attempt to descend in directions that run the tendons into slack, and, conversely, avoids that tendons are run into slack due to joint motion.

To enforce unidirectionality constraints while maintaining continuity and global convexity, we add a quadratic constraint potential term *c*(**p**), given by:

(26) $$c\left(\text{p}\right)=-\gamma_{\mathrm{const}}{∥\Delta^{-}\left(\text{p}\right)∥}_{2}^{2},$$

where **Δ**^−^(**p**) = min(**Δ**(**p**), 0) is the element-wise minimum, i.e., the constraint is only active for branches that are currently in slack. $\gamma_{\mathrm{const}}\in R$ is a large scaling constant. By adding the constraint potential gradient, $\dot{\text{p}}$ is given by

(27) $$\dot{\text{p}}=-\gamma_{e}\;\nabla_{\text{p}}{∥\text{e}∥}_{2}^{2}+\nabla_{\text{p}}\;c\left(\text{p}\right),$$

where $\nabla_{\text{p}}{∥\text{e}∥}_{2}^{2}$ is given by (24) and $\nabla_{\text{p}}\;c\left(\text{p}\right)=-2\gamma_{\mathrm{const}}\Delta^{-}\left(\text{p}\right)$. Similar to (25), the second-order gradient of *c*(**p**) results in a quadratic form which is globally convex. This constraint replaces the slack control component of the control strategy described in Roozing et al. (2016). Achievable values of **p** due to mechanical constraints can be similarly imposed in a convex manner.

#### 4.2.2 Compensating for $\dot{\text{q}}\neq$ 0

To ensure the convergence of the error under non-zero joint motion, we extend the above gradient descent based control law with an additional term taking this motion into account. Given $\dot{\text{q}}$, we solve $\dot{\text{e}}=0$ for $\dot{\text{p}}$ in (22):

(28) $$\begin{array}{lll} 0 & = & -\left(\nabla_{\text{p}}\;\tau \right)\dot{\text{p}}+\left(\nabla_{\text{q}}\;\tau^{*}-\nabla_{\text{q}}\;\tau \right)\dot{\text{q}}\\ \left(\nabla_{\text{p}}\;\tau \right)\dot{\text{p}} & = & \left(\nabla_{\text{q}}\;\tau^{*}-\nabla_{\text{q}}\;\tau \right)\dot{\text{q}}\\ \dot{\text{p}} & = & {\left(\nabla_{\text{p}}\;\tau \right)}^{-1}\left(\nabla_{\text{q}}\;\tau^{*}-\nabla_{\text{q}}\;\tau \right)\dot{\text{q}}\\ & = & -{\left(T^{T}K\right)}^{-1}\left(T^{T}K\;T+\nabla_{\text{q}}\;\tau^{*}\right)\dot{\text{q}}, \end{array}$$

which we will refer to as ${\dot{\text{p}}}_{\mathrm{dq}}$. This yields the rate of change of **p** needed to compensate for the change in **q**, and thus keep the error constant. The first term is equal to $-T\;\dot{\text{q}}$, and simply ensures that **p** + *T* **q**, i.e., the elongation **Δ**, remains constant. The second term is equal to $-{\left(T^{T}K\right)}^{-1}\left(\nabla_{\text{q}}\;\tau^{*}\right)\dot{\text{q}}$ and compensates the change in desired torque due to $\nabla_{\text{q}}\;\tau^{*}\neq \text{0}$. Of course, this last term requires knowledge of how the desired torques will change as the joint configurations change and is generally not trivial to implement. Combining (28) with (27):

(29) $$\dot{\text{p}}=-\gamma_{e}\;\nabla_{\text{p}}{∥\text{e}∥}_{2}^{2}+\nabla_{\text{p}}\;c\left(\text{p}\right)+\gamma_{\mathrm{dq}}\;{\dot{\text{p}}}_{\mathrm{dq}},$$

we obtain the rate of change of **p** that results in global asymptotic convergence of **e**. The scaling constant 0 ≤ *γ_dq_* ≤ 1 avoids excessive adjustment of the pretension to compensate the joint motion, which for high gear ratios may reduce energy efficiency, and is dependent on the mechanical implementation of the actuators.

#### 4.2.3 Computing the Adjustment Velocities

The rate of change of **p** given by (29) may not be achievable in practice due to speed limitations following from the mechanical implementation. Hence, $\dot{\text{p}}$ is scaled as follows to obtain the reference adjustment velocity ${\dot{\text{p}}}^{*}$:

(30) $${\dot{\text{p}}}^{*}=\alpha \;\dot{\text{p}},$$

where

(31) $$\alpha =\left\{\begin{array}{cc} \frac{p_{\mathrm{vmax}}}{\text{max}\left(\left|\dot{\text{p}}\right|\right)} & \text{max}\left(\left|\dot{\text{p}}\right|\right)>p_{\mathrm{vmax}}\\ 1 & \text{Otherwise} \end{array}\right.,$$

and *p_vmax_* denotes the maximum achievable adjustment velocity. This ensures none of the branches are commanded beyond their speed limit, which would result in not descending the gradient of the error norm in the correct direction.

### 4.3 Rankedness of *T*

The case of *T* not being full rank has one important consequence; the solution is redundant. An intuitive interpretation of this is the example of two antagonistic branches driving a single joint, in which increasing the tension of both in a certain proportion (given by their relative transmission ratio and stiffness values) does not result in a change of net torque. This is an example of a single rank deficiency of *T*, resulting in a line in the **p** configuration space providing identical joint torques. For more complex systems, *T* may be multiple rank deficient.

Since optimal energy efficiency is obtained by minimizing the tension throughout the system that loads the pretension mechanisms, a unique solution may be obtained in the null space of the obtained solution. In the following, we suggest two extensions toward this end.

#### 4.3.1 Pseudoinverse

When using the pseudoinverse based pretension control of Section 4.1, the following extension may be used, minimizing the squared *l*_2_-norm of the deflections **Δ** in the null space of the solution of (20):

(32) $$\begin{array}{l} \underset{\text{x}}{\min}{∥\Delta ∥}_{2}^{2}={∥{\text{p}}_{\mathrm{psdo}}+Z\text{ x}+T\text{ q}∥}_{2}^{2}\\ \;\text{s}.\text{t}.\Delta \geq 0\;\text{and}\;\underline{p}\leq {\text{p}}_{\mathrm{psdo}}+Z\text{ x}\leq \bar{p}, \end{array}$$

where *Z* = ker (−*T^T^ K*), **p***_psdo_* denotes the pseudoinverse solution for p* given by (20), and $\underline{p}$, $\bar{p}$ denote the lower and upper bounds on **p**, respectively. Given a solution **x***_opt_* of (32), the new value for the desired pretension positions p* is given by **p***_psdo_* + *Z* **x***_opt_*.

#### 4.3.2 Gradient Descent

For the gradient descent based solution of Section 4.2, one may add a gradient term *c_tens_* (**p**):

(33) $$c_{\mathrm{tens}}\left(\text{p}\right)=-\gamma_{\mathrm{tens}}{∥\Delta \left(\text{p}\right)∥}_{2}^{2},$$

for which the gradient w.r.t. **p** is given by $\nabla_{\text{p}}\;c_{\mathrm{tens}}\left(\text{p}\right)=-2\gamma_{\mathrm{tens}}\Delta \left(\text{p}\right)$. This gradient is then added to (29). For simplicity and illustration of the core ideas of this work however, we shall focus on systems with full rank *T* for the remaining sections.

### 4.4 An Illustrative Example

**Figure 4:**
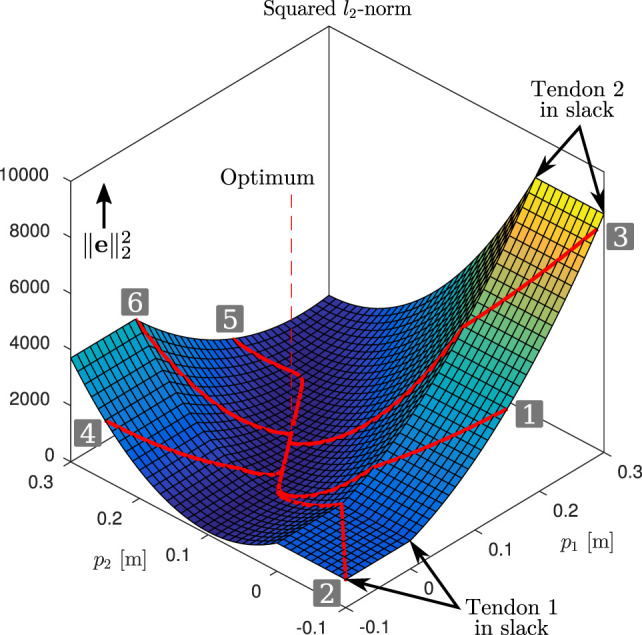
Gradient descent: squared *l*_2_-norm of e. The superimposed red lines show example evolutions (see also time evolutions in Figure 5) of p and the corresponding squared *l*_2_-norm of the error. They can be seen to converge to the global minimum, indicated by the vertical dashed line.

To illustrate the core ideas behind the gradient descent based control law, we start with a simple example of a biarticulated robot with two joints and two adjustable compliant tendons in a static configuration $\left(\dot{\text{q}}=0\right)$. The actuation topology is described by

(34) $$T=\left[\begin{array}{c} {\text{t}}_{1}\\ {\text{t}}_{2} \end{array}\right]=\left[\begin{array}{cc} -0.1 & -0.2\\ 0 & 0.3 \end{array}\right],$$

i.e., the first tendon is biarticulated, and the second is monoarticulated. The first joint is driven only by the first tendon, and the second joint is driven by both tendons in an antagonistic manner. We assume the tendons to be unidirectional. The stiffness matrix *K* is given by *K* = diag(1000,1000), and the joint configuration **q** = [0,0]*^T^*. The reference torques are set to τ* = [10, −30]*^T^* Nm in this example. Furthermore, we set the constraint parameter *γ_const_* = 10^8^ and gradient descent parameter *γ_e_* = 5 × 10^−6^. Lastly, we assume a maximum adjustment velocity of *p_vmax_* = 0.05 m/s. The landscape of the squared *l*_2_-norm is shown in Figure 4, together with six example evolutions with varying initial conditions for **p**. They can be seen to all converge to the global minimum, indicated by the vertical dashed line.

**Figure 5:**
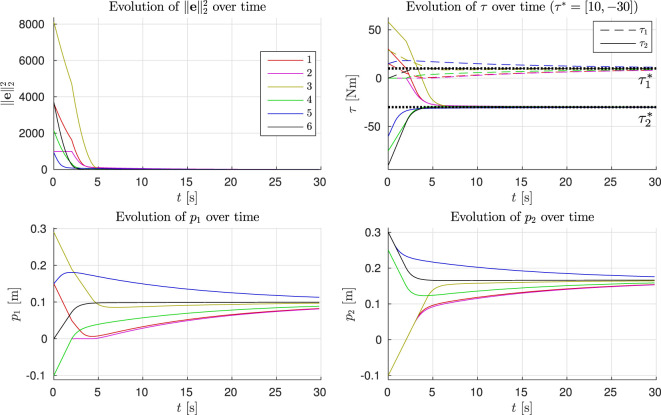
Time evolution of the six evolutions shown in Figure 4.

The time evolutions of ${∥\text{e}∥}_{2}^{2}$, ***τ***, and **p** are shown in Figure 5. As the desired torques can be achieved with **Δ** ≥ **0** and *T* is full rank, the error norm converges to zero for all evolutions. One can observe that while **p** takes relatively long to converge (bottom figures), this is beneficial: the error norm is very small after 5 s (top-left figure), and further adjustment of the pretension yields only small reduction of the error. Out of these six example evolutions, numbers 1–4 have initial conditions where at least one of the two branches is in slack. It can be seen that the constraint described in Section 4.2.1 is effective, driving the branches out of slack at the maximum velocity. From the time evolutions of *p*_1_ and *p*_2_ (bottom figures), one may be tempted to think there is undesired overshoot in the pretension positions (e.g., evolution 1). However, this “overshoot” is desired, as due to the biarticular coupling between the joints, this reduces the torque error norm while the other pretension position converges.

## 5 Simulation Study

In this section, we present a simulation study on the model of a planar 3-DoF leg prototype which was recently developed Roozing et al. (2018). The prototype follows the same design concepts as in Roozing et al. (2015) and Roozing et al. (2016), with all joints driven by torque-controlled series-elastic actuators, augmented with parallel compliant branches with adjustable pretension. The model includes viscous friction components at the joints, actuator dynamics with friction in the motor drives and drive trains, and elastic element internal damping. Furthermore, low-level torque control is implemented for the SEAs, velocity control is implemented for the parallel pretension motors, and voltage and current limits are imposed. For more details on their dynamics modeling, we refer the reader to Roozing et al. (2016). The design features three actuated degrees of freedom: ankle, knee and hip, and is semi-anthropomorphic, with similar mass and mass distribution to the human limb. The trunk link is loaded with an additional 20 kg, simulating the weight of a full humanoid in two-legged stance.

**Figure 6:**
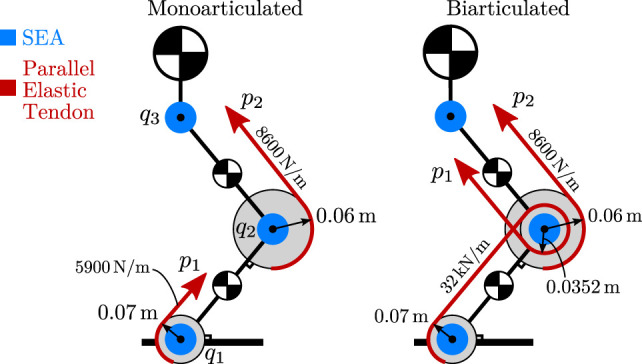
3-DoF leg model used in simulation, shown in both mono- and biarticulated actuation configurations.

A diagram of the model is shown in Figure 6. In this case, two actuation topologies are considered; one that includes two monoarticulated parallel elastic branches on knee and ankle, and one where one of the two branches is biarticulated, spanning the ankle and knee joints. The design parameters were optimized following the procedure outlined in Section 3, and the actuation topology matrices are given by

(35) $$\begin{array}{lll} T_{\mathrm{mono}} & = & \left[\begin{array}{ccc} -0.07 & 0 & 0\\ 0 & 0.06 & 0\\ 0 & 0 & 0 \end{array}\right],\\ T_{\mathrm{bi}} & = & \left[\begin{array}{ccc} -0.07 & -0.0352 & 0\\ 0 & 0.06 & 0\\ 0 & 0 & 0 \end{array}\right], \end{array}$$

and the stiffness matrices are given by *K_mono_* = diag(5900,8600,0) and *K_bi_* = diag(5900,8600,0), respectively. As evidenced by the zero columns in (35), the hip joint is not augmented with a parallel branch.

**Figure 7:**
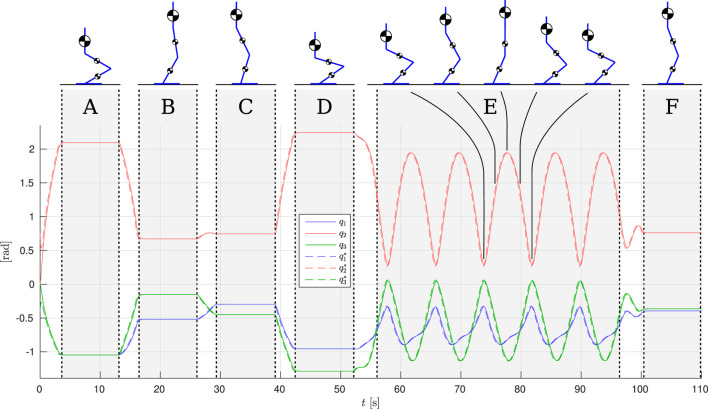
Joint references and tracking.

In this study, we first perform a number of point-to-point motions in Cartesian space with the hip of the robot, keeping the torso upright. Each pose is maintained for 10 s to clearly illustrate the transient behavior of the proposed control strategy given the system’s parameters. Figure 7 shows the joint configuration references and tracking for the biarticulated configuration; the monoarticulated configuration is not shown for brevity, however, tracking is almost identical. The figure is augmented to show the leg poses at various time instances, showing the wide range of executed motions. Phases A–D and F denote the aforementioned static poses. The second part of the reference motion involves a cyclic Cartesian trajectory of the hip in an elliptical squatting motion, to demonstrate its behavior under highly dynamic motion. This part is denoted as phase E in Figure 7.

The robot is controlled with simple joint-level impedance controllers as high-level control strategy, providing the torque references for the gradient descent based controller of Section 4.2. As in Roozing et al. (2015) and Roozing et al. (2016), the SEAs are torque controlled to ensure the desired net torques are always achieved at the joints. We set the gradient descent parameter *γ_e_* = 1 × 10^−6^, the constraint parameter *γ_const_* = 10^2^, and *γ_dq_* = 0.1. The maximum pretension adjustment velocity of this system is approximately 3 cm/s, imposed by the transmission ratio, chosen electric motors and supply voltage of 48 V.

**Figure 8:**
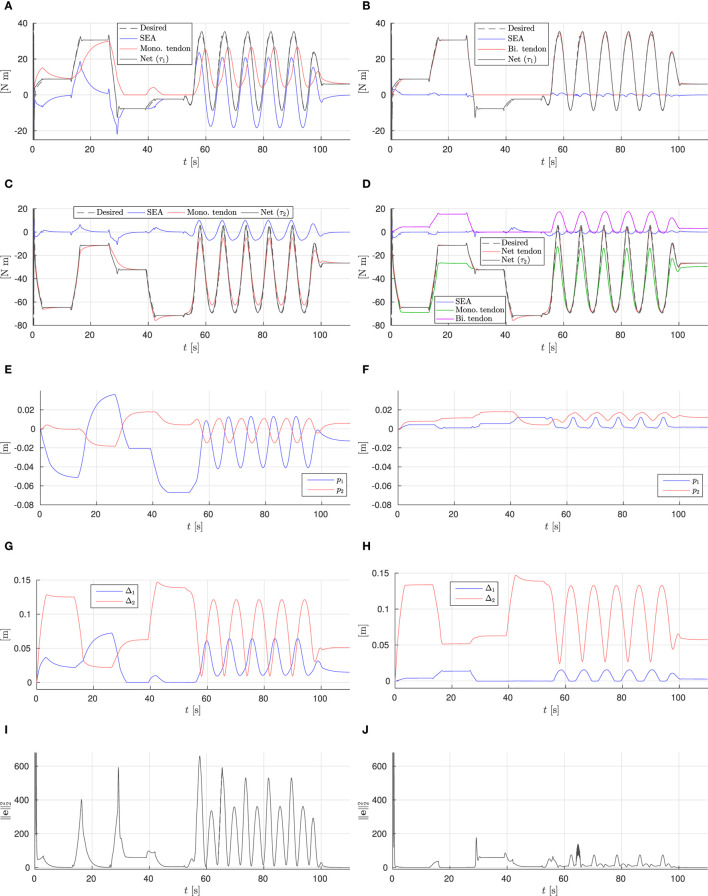
Simulation results. **(A)** Monoarticulated: ankle (q1) torques. **(B)** Biarticulated: ankle (q1) torques. **(C)** Monoarticulated: knee (q2) torques. **(D)** Biarticulated: knee (q2) torques. **(E)** Monoarticulated: pretension positions. **(F)** Biarticulated: pretension positions. **(G)** Monoarticulated: elastic element elongation. **(H)** Biarticulated: elastic element elongation. **(I)** Monoarticulated: squared l2-norm of error. **(J)** Biarticulated: squared l2-norm of error.

The results are shown in Figures 7 and 8. The torque plots for the ankle (Figures 8A,B) and knee (Figures 8C,D) confirm that indeed the net torques ***τ***_1_ and ***τ***_2_ are nearly identical when comparing the mono- and biarticulated cases, showing that the SEAs can effectively ensure the desired net torque is achieved at the joints, and that the motions are comparable.

Considering on the torque provided by the parallel elastic tendons (red lines in Figures 8A–D) for both joints in both cases, they can be observed to converge to the net desired torque, causing the required SEA torque to converge to zero, unless the desired joint torque is not feasible given the tendon actuation topology. For example, negative ankle torques cannot be provided by the ankle tendon, causing the tendon torque to converge to zero and the SEA providing the full negative torque (e.g., phases C and D, where the center of pressure is behind the ankle joint and the ankle needs to provide negative torque). Furthermore, from the elastic element elongation shown in Figures 8G,H, it can be observed that the constraint (Section 4.2.1) effectively constrains the unidirectional tendons to zero elongation. These results show the gradient descent based control approach is effective at achieving torque control of the system using (multi-)articulated compliant arrangements.

During the cyclic motion part of the reference, the tendon torques are unable to converge to the reference torque entirely, as the pretension adjustment speed limits do not allow for it (and the load motion compensation parameter *γ_dq_* = 0.1); however, their smaller adjustments combined with the optimized design do lead to a substantial reduction of the error, causing the SEAs to need to deliver only a fraction of the net joint torque. This in turn allows to design for small, light, efficient motors. In the monoarticulated knee case, the SEA is providing less than 10 Nm peak torque out of approx. 70 Nm required net peak torque. In the biarticulated case, the SEAs are providing less than 5 Nm on both the knee and ankle joints. In the monoarticulated ankle case, a smaller reduction in torque requirements is observed; the dependence of ankle load on the configuration of both joints results in the monoarticulated tendon not providing a torque that matches well with the required torque, despite substantial pretension adjustment of the ankle tendon (Figure 8E).

Comparing the two actuation topologies, we observe that the biarticulated configuration is both able to provide the desired net joint torques more accurately, as well as needing significantly smaller pretension adjustments to achieve them. This conclusion is further strengthened by comparing the squared *l*_2_-norm of the error for both cases, shown in Figures 8I,J. We can therefore conclude that the biarticulated configuration is more suitable for the system under consideration.

## 6 Conclusion and Future Work

This work has developed a novel model formulation of compliant actuation structures for articulated robots, including multiple branches and multiarticulation. The modular formulation employs a single matrix to describe the entire actuation topology, and formulates the energy exchange between the compliant elements and articulated multibody robot dynamics using N-D power flows.

Using this formulation, we derived a novel gradient descent based control law for compliant actuation structures with adjustable pretension, with proven convexity for arbitrary actuation topologies. Unidirectional elastic elements were considered through the inclusion of a convex constraint into the formulation.

A simulation study on a 3-DoF leg model using two different actuation topologies demonstrated that the gradient descent based control method is effective for torque control of the parallel tendons, leading to asymptotic convergence of the error. Additionally, the results illustrate that the chosen actuation topology and optimization of its design parameters are also fundamental for optimal performance.

We believe this control strategy is promising, and future work will include the application of this strategy to the 3-DoF hardware prototype, which is currently under development and will allow for rapid interchange of several actuation topologies, including those considered in this work. In terms of future work, the proposed formulation lends itself very well to the inclusion of energy expenditure; the magnitude of pretension adjustment can be considered in the context of energy consumed by the motors to do so. Furthermore, whereas in the presented simulation study series-elastic main drives were augmented with parallel elastic tendons, we believe effective systems can be designed that employ only such elastic tendons, in multiarticulated configurations, similar to the human anatomy. Lastly, extensions toward predictive control in an energy efficiency context are promising.

## Author Contributions

This work was fully performed by WR.

## Conflict of Interest Statement

The author declares that the research was conducted in the absence of any commercial or financial relationships that could be construed as a potential conflict of interest.
